# Cytoskeleton Response to Ionizing Radiation: A Brief Review on Adhesion and Migration Effects

**DOI:** 10.3390/biomedicines9091102

**Published:** 2021-08-28

**Authors:** Giuseppe La Verde, Valeria Artiola, Valeria Panzetta, Mariagabriella Pugliese, Paolo A. Netti, Sabato Fusco

**Affiliations:** 1 Istituto Nazionale di Fisica Nucleare, INFN Sezione di Napoli, 80126 Naples, Italy; glaverde@na.infn.it (G.L.V.); pugliese@na.infn.it (M.P.); 2 Dipartimento di Farmacia, Università degli Studi di Napoli Federico II, 80131 Naples, Italy; 3 Centro Servizi Metrologici e Tecnologici Avanzati, Università di Napoli Federico II, 80146 Naples, Italy; val.artiola@gmail.com; 4 Centro di Ricerca Interdipartimentale sui Biomateriali (CRIB), Dipartimento di Ingegneria Chimica, dei Materiali e della Produzione Industriale, Università degli Studi di Napoli Federico II, 80125 Naples, Italy; nettipa@unina.it; 5 Centre for Advanced Biomaterial for Health Care, Istituto Italiano di Tecnologia, 80125 Naples, Italy; 6 Dipartimento di Fisica “Ettore Pancini”, Università degli Studi di Napoli Federico II, 80126 Naples, Italy; 7 Dipartimento di Medicina e Scienze della Salute “Vincenzo Tiberio”, Università del Molise, 86100 Campobasso, Italy; sabato.fusco@unimol.it

**Keywords:** mechanobiology, cytoskeleton, cancer, ionizing radiation, adhesion, migration

## Abstract

The cytoskeleton is involved in several biological processes, including adhesion, motility, and intracellular transport. Alterations in the cytoskeletal components (actin filaments, intermediate filaments, and microtubules) are strictly correlated to several diseases, such as cancer. Furthermore, alterations in the cytoskeletal structure can lead to anomalies in cells’ properties and increase their invasiveness. This review aims to analyse several studies which have examined the alteration of the cell cytoskeleton induced by ionizing radiations. In particular, the radiation effects on the actin cytoskeleton, cell adhesion, and migration have been considered to gain a deeper knowledge of the biophysical properties of the cell. In fact, the results found in the analysed works can not only aid in developing new diagnostic tools but also improve the current cancer treatments.

## 1. Introduction

Mechanobiology focuses attention on the relations between inborn and outborn physical forces, structure and mechanics of nucleus and cytoskeleton (CSK), and biological processes, such as cell proliferation, gene expression, or disease development [1,2]. Specifically, this emerging science is continuously offering new experimental and computational tools for studying and better understanding the importance of CSK in the emergence and progression of many diseases. The CSK is one of the main components of all cells. Situated in the cytoplasm, it is composed of thereby three major components, each of which plays a specific role in several biological processes: microtubules, actin filaments, and intermediate filaments [3,4]. In detail, microtubules are involved in controlling the cellular shape, cell transport, cell motility, and cell division processes [5,6,7]. Actin filaments, composed of filamentous (F) and globular (G) proteins, play an important role in endocytosis [8], exocytosis [9], and mechanical stability [10]. Finally, intermediate filaments, which are the least rigid structure of the CSK [11,12], have the capability of anchoring organelles into the cell and are therefore deemed mechanical buffers [13,14]. Furthermore, they are involved in several other processes, such as the modulation of mitochondrial motility [15]. Intermediate filaments are also involved in cell-extracellular matrix (ECM) crosstalk [16], cell migration and adhesion [17,18], and in controlling cortical and intracellular cell stiffness [19]. Nonetheless, the CSK has been identified as a leading player in a large plethora of both physiological and pathological processes, such as cell adhesion [20] and migration [21].

Many studies have shown the strict connection between the alteration of the cytoskeletal architecture and the development of various diseases. In fact, several pathologies have been associated with aberrations of the cytoskeletal proteins or changes in the CSK structure. For instance, while the development of various neurodegenerative diseases involves the mutations of cytoskeletal genes, others require alteration in the cytoskeletal structure. In particular abnormalities in the actin filaments can damage the neurite growth or lead to an anomalous formation of dendritic spines and synapses. Microtubules play a fundamental role in neurodegenerative diseases too. In fact, disruptions in this specific CSK component can lead to both the formation of retraction bulbs and axonal retraction [22,23,24]. It has been proven that the CSK is involved in liver diseases and myopathies too. In the former, alteration in keratin proteins, which are a subfamily of the intermediate filaments, can cause a disruption of the intermediate filaments network in the liver [25]. Conversely, actin is the most affected structure in myopathy. Variations in the G-actin can alter the functions and stability of the F-actin which, in turn, can affect the hydrolysis and binding of nucleotides, leading to the development of the disease. In addition, it has been observed that alterations in the F-actin, in particular its decrease, are strictly correlated to a reduction of both cellular stiffness and migratory abilities [26]. In addition to this, an increase in the density of both the microtubules and the intermediate filaments in heart failure has been observed [27]. Other studies have shown that the CSK is involved in dendritic cells (DCs) maturation, which activates T cells, through the remodeling of the actin filaments [28]. This CSK alteration leads to an increase in the cell’s stiffness which, in turn, promotes T cells priming [29,30].

Even more specifically, many studies have proven how cytoskeletal abnormalities play a fundamental role in cancer onset and progression [31,32]. In particular cancer cells show some alterations, both morphological and phenotypical, which suggest that actin filaments are to be considered fundamental in the malignant transformation. For instance, the cytoplasmic actin depolymerization, which can be determined by the increase in the G-actin and the simultaneous decrease in the F-actin, can be used as a marker for cancer detection [33]. In addition to this, during the processes of transformation from a normal cell to a cancerous one, the architecture of the CSK becomes irregular and less rigid, effectively impacting the adhesiveness and increasing the migratory and invasive abilities of cells [34,35,36]. In most cancers, the process of invasion is directed by different types of migration, which are observed simultaneously [37]. In fact, cells can move together in a sheet-like structure, a process called collective migration [38,39], or an individual cell can separate from the others and migrate alone, a practice called the individual migration [40]. However, cells are systems that communicate with the external environment by adapting responses to optimize their migration process. For this reason, the collective and individual migration are in dynamic alternation [41,42]. A very significant sign of tumour invasion and progression is the epithelial-mesenchymal transition (EMT) process that is also involved in tumour initiation, metastasis formation, and resistance to therapy [43,44,45]. In the EMT process, epithelial cells, which are polarized and non-motile, dispel the cell-cell junctions, display an altered adhesiveness, and become motile, non-polarized, and invasive mesenchymal cells. This switch can lead to an increase in the migratory and invasive tendency of cells, due to the modulation of growth factor signalling and the remodelling of the actin CSK. EMT is induced by several factors, such as gene mutations or growth factor signalling, and the cancerous cells that go through this process can control different biological activities, which are essential to the behaviour of the cell [46].

Additionally, the CSK is involved in both migration and adhesion processes as a response to changes and stresses coming from the tumour microenvironment (TME), which is the result of the interaction among tumour cells, stromal cells, and the ECM [47]. Through the mechanotransduction process, the CSK converts physical stress into a biochemical response, affecting the behaviour of the cells (e.g., division, adhesion, migration) [2]. Mechanical stimuli are therefore picked up and sent to the cells through the activation of surface mechanosensors such as integrins [48], TRP ion channels [49], and YAP/TAZ molecular complex [50].

It is known that both the CSK and the ECM are essential to the correct functioning of the tissue since their alteration can lead to tumorigenesis. In fact, from one side, as already discussed, cancerous cells are characterized by a less structured CSK, lower mechanical and cyto-adhesive properties compared to their corresponding normal, healthy cells and this higher cancer cell deformability has been discussed as a method to enhance the ability to penetrate tissues and metastasize to distant sites, overcoming physiological barriers posed by confined spaces within the ECM and capillary walls. On the other side, an opposite trend in the alteration of the mechanical microenvironment and cell mechanics, during tumour transformation and progression, has been reported. In particular, while cells undergo a softening stage, their ECM experiences a stiffening stage supporting the hypothesis of the regulatory function of the ECM along the tumorigenesis and tumour progression [32]. The importance of CSK and ECM is becoming clearer also in mediating the response to various therapies to the point that they are now considered one of the targets of different cancer treatments [51].

In particular, ionizing radiation can affect several biological processes, such as cell adhesion and migration, through tissue stiffening. For instance, various works determined an increase in the invasiveness of both cancerous cell lines MDA-MB-231 and MCF-7 after the radiation treatment due to the physical crosslinking of collagen [52,53]. Additionally, tissue stiffening can also reduce cell adhesion, leading to an enhancement in cell dissemination [54].

For these reasons, the evaluation of the CSK in response to classical therapeutic approaches, such as chemotherapy and radiotherapy, is potentially useful to obtain new and complementary information for the optimization process of their therapeutic outcomes. One of the most effective cancer treatments is radiotherapy and the conventionally used technique is external beam radiotherapy (EBRT). With this system, the radiation, that is photons produced by an external source such as a high-energy accelerator, is delivered in fractionated doses, which are usually equal to 2 Gy for five days a week [55].

Radiation oncology is based on the 4Rs of radiotherapy, which are repair, redistribution, repopulation, and reoxygenation. A fifth R is usually added to these—radiosensitivity, that is the early DNA damage produced by the radiation. Based on these concepts, conventional radiotherapy is developed to deliver the maximum dose to the tumour while sparing the normal tissue. To do that, the tumoral site is initially localized through computed tomography (CT) or magnetic resonance imaging (MRI). These technologies allow the development of several models which aid in obtaining an optimal dose delivery plan [56].

EBRT is the most commonly used form of radiation oncology treatment, but there are other procedures developed for this purpose, such as brachytherapy or FLASH therapy. The former is based on the concept of implanting radioactive “seeds” close to the tumoral site to deliver a high dose of radiation directly to the cancer cells, effectively sparing the normal tissue [57]. Conversely, in FLASH radiotherapy the delivered dose is in the range of 10–20 Gy with a dose rate of 50 Gy/s. Recent experiments have shown that through this practice there is a noticeable decrease in tissue toxicity at a high dose rate, effectively improving the radiotherapy treatment [58].

Many studies have investigated both direct and indirect effects of ionizing radiations on the cell. The most well-documented study in the literature is radiation-induced DNA damage. The 30–40% of lesions to the DNA molecule are due to the direct effect of ionizing radiation, while the rest is given by the generation of free radicals which can harm the DNA [59]. These damages can lead to several outcomes, ranging from mutation, carcinogenesis, or cell death to cell recovery [60,61,62]. Worth mentioning are the studies conducted by Woloschack and colleagues, where they analysed the radiation-induced mutations of the genes encoding the cytoskeletal elements [63,64,65]. In particular, while one of their studies have proven that the mRNA for beta-actin was repressed after the exposure to X-rays [63], others have demonstrated the alterations in mRNA expression of three CSK and ECM elements, namely tubulin, actin, and fibronectin [64,65]. In fact, during the first hour after exposure, it was possible to observe the accumulation of α-tubulin and γ-actin and the reduction in the expression of β-actin mRNA. It was proven that the accumulation of transcripts for these genes increased in a dose-dependent manner [64,65].

More recently, research on radiation-induced effects on cells has shifted to mechanobiology aspects and focused on how DNA damages can affect physical forces and the mechanical integrity of cells. However, the scientific literature lacks a systematic and comprehensive analysis of the role of radiation in cell mechanobiology and, given the rising importance of CSK dynamics in controlling tissue physiopathology, this brief review will focus on the alterations of the cytoskeletal proteins and the related cells’ functions, such as adhesion and migration, after the therapeutical delivery of ionizing radiations.

## 2. Radiation Effects on the Actin CSK

As previously stated, ionizing radiation can produce several effects on the CSK. Whilst some studies reported that radiation treatment stiffens the actin filaments, others have reported the opposite phenomenon. In the following paragraphs, both effects will be reviewed and discussed.

### 2.1. Ionizing Radiation and the Increase in the Polymerization of the Actin Filament

The thickening of the CSK as an effect of ionizing radiation was the subject of many studies. In particular, it is worth mentioning research performed on two melanoma cell lines: Mel270, which is a uveal melanoma cell line, and BLM, a cell line that originated from the metastasis of skin melanoma [66]. The authors observed that both cell lines did not show changes in the total level of actin protein, but important rearrangements of the actin CSK. Specifically, both Mel270 and BLM cell lines, after irradiation, exhibited a significant thickening in the marginal actin fibres, accompanied by a reduction of the internal ones, also known as stress fibres. These alterations to the actin CSK were identifiable for a long time from irradiation (~40 days) and were deemed responsible for the lowering of the cells’ elastic modulus (or Young’s modulus) [66]. The authors speculate that these effects can be mediated by the activation of the RhoA/ROCK1 signalling pathway, which has a key role in controlling actin stress fibres formation, cell contractility and VE-cadherin adherens junction redistribution [67], as previously observed in endothelial cells [68]. Another study, performed on BALBc/3T3 and SVT2 cells, proved that using doses of 1 Gy and 2 Gy of X-rays affected cell morphology and actin CSK [69,70]. In particular, the radiation treatment promoted the actin polymerization which stimulated the thickening of the structure and, consequently, led to an increase in the focal adhesions (FAs) areas. The FAs, in fact, constituting the structural elements that physically connect the actin CSK to the external microenvironment, mediate many relevant processes, among which the cytoskeletal organization and remodelling [71]. As a result, 24 h after the delivery of the doses both cell lines displayed an increase in the actin filaments in the CSK and a growth in their spreading area. In particular, both cell lines showed an increase in the expression of α-actin, a CSK protein, which is linked to the augmentation of cell adhesiveness and stiffness [69,70]. Conversely, Mohammadkrim et al. investigated the effects of fractional radiotherapy doses (2 Gy up to 8 Gy) on the CSK, using human umbilical vein endothelial cells (HUVECs) [72]. After the treatment, cells showed an increase in their stiffness, alteration induced by both the reorganization of the CSK and the increase in the nucleus area. Specifically, the remodulation of the cell mechanical properties was considered associated with a relocation of the F-actin fibres under the cellular membrane. The authors speculated that the increase in the nucleus area could be due to the arrest of cells in the G2 phase [73], where cells and nuclei result to reach their largest sizes [74] and considered this nuclear expansion responsible for the compression applied to the actin fibres under the cell cortex, the growth of the cellular resistance to deformation and then, the higher cellular stiffness [72]. However, it is important to consider that the augmentation of cytoskeletal stiffness could be not the direct consequence of the nuclear expansion, but the cause of this phenomenon. In fact, contractile actin structures, thanks to the LINC complex (Linker of Nucleoskeleton and CSK), can directly transmit mechanical forces to the nucleus and the genome at its interior, change its shape and regulate gene expression [75]. In particular, it has been demonstrated that a strict correlation exists between CSK organization, cell spreading and nuclear shape [76], indicating that the increased content of F-actin and the cell stiffening could be responsible for changes in nuclear shape. Many other studies focused their attention on the effects of single-dose X-rays radiation on the CSK properties. One of these findings reported the alterations in the cytoarchitecture of two glioblastoma cell lines, LN229 and U87 [77]. These changes involved the activation of the small GTPases Rac1, which led to the inactivation of RhoA. As previously mentioned, both these proteins are implicated in the organization of the actin filaments and the development of the adhesion sites [78]. Indeed, after the exposure to ionizing radiation, the U87 cell line showed a higher density in the CSK actin filaments than the other cell line. In addition to this, the analysis of the actin CSK revealed a modification in the actin structure after the radiation treatment. These changes, which led to an increase in cells’ stiffness, were hypothesized to be produced by the decrease in the G-actin and the increase in the F-actin amount, and/or by an altered dynamics of actin CSK polymerization, and/or by a different activity of cross-linkers [77].

The radiation treatment can produce several structural changes, in a dose-dependent manner, in many other types of cells. Among these, worth mentioning are the cortical neurons. In fact, X-ray radiation has the potential to generate several alterations to CSK proteins, causing morphological changes [79] that can ultimately lead to neuronal death since actin filaments and microtubules play a fundamental role in the early stages of apoptosis [80,81]. Not to mention, radiation causes both the decomposition and the rearrangement of the neural cells’ CSK, with particular regard to the F-actin. These effects usually generate the alterations of the skeletal proteins which lead to the disruption of the cell membrane and a dense redistribution of the CSK in the perinuclear region and a relevant increase in cell stiffness [79]. The authors focused their attention principally on the alteration of microtubules’ structure, but they speculated on the involvement of actin CSK to explain the stiffening effect observed after irradiation.

### 2.2. Ionizing Radiations and the Decrease in the Expression of the Actin Filament

Many other studies have proven that ionizing radiations have an inhibitory effect on the polymerization of the actin filaments. For instance, more than a few groups of researchers have reported this effect after the delivery of low dose X-ray radiation (<1 Gy), which affects cell structures in different ways. Low doses of radiation have the ability not only to affect different molecular mechanisms, such as DNA double-strand breaks, and the formation of oxygen species [82,83,84] but also to influence the reorganization of the CSK and the alteration of the cell morphology. These changes can impact some of the biological processes, such as proliferation and differentiation [85,86]. Other studies reported how actin networks of endothelial cells were impaired after the radiation treatment [87], while yet another research demonstrated how human respiratory epithelial cell lines reacted after the delivery of X-rays [88]. In particular, the results of this research showed an increase in F-actin depolymerization.

Huang et al. examined the alterations in the CSK of osteoblasts after the delivery of 0.5 Gy X-ray radiation [89]. Notably, the study reported changes in the structure of intracellular actin. These effects were observable for 5 days after the delivery of the X-ray dose, effectively implying these changes to be induced by ionizing radiation. In addition to this, a decrease in the expression level of the F-actin was observed. Furthermore, the authors investigated the expression of proteins, such as RhoA, ROCK1, and p-cofilin, which stimulate the reorganization of the CSK due to actin depolymerization [90]. These proteins were involved in the reorganization of the actin filaments after the delivery of radiation, effectively proving that they manage the dynamics behind the regulation of the actin CSK [89].

The effects of low doses of X-rays on the CSK were also studied using murine exorbital lacrimal gland cells, which were irradiated with a dose of 36 mGy. The analysis showed that following irradiation the actin filaments in the cell cortex exhibited depolymerization with a consequent increase in the cellular area. Alterations of the actin microfilaments can lead to intracellular changes which can affect several cell functions. In addition to this, it was observed that low doses of radiation were able to modify both actin and intermediate filaments [91]. Nonetheless, these outcomes were shown to be reversible, since the cells returned to the control condition 24 h after the treatment [92].

Even though evident alterations in CSK elements and gene expression are reported when low doses of radiation were used, they are probably the sequelae of interference with cell control processes that result in temporary or permanent shifts in cell characteristics, rather than actual effects of radiations on the CSK. In fact, the low dose radiation-induced DNA damages are generally much less relevant than the damages caused by the oxidative processes of metabolism and the repair mechanisms carried out by the cell are effective at low radiation.

The alterations of the CSK structures have been studied also with the employment of higher doses, which are utilised in conventional radiotherapy treatment. For instance, Zheng et al. studied the effects of different doses (0–4 Gy) on the CSK using tongue squamous cell carcinoma (TSCC) cells [93]. The results showed that, after the treatment with increasing doses of X-rays, TSCC cells displayed gradual disorganization of the F-actin network and a consequent decrease in Young’s modulus [93,94]. As previously mentioned, BLM cell lines, after irradiation, showed a substantial reduction of the internal actin fibres and a consequent dose-dependent CSK softening [66]. Mel270 cells manifested a similar alteration in the CSK structure, but no variation in cell mechanical properties was detected 20 days after irradiation [66]. To explain this unexpected finding, it is important to consider that, even though cell mechanical properties are mainly dominated by the actin CSK (F-actin/G-actin ratio, length and thickness of actin stress fibres, contractile cortical network, etc.), other CSK constituents, such as microtubules and intermediate filaments, can be impaired by radiation and their role needs to be considered in future investigations to have a comprehensive understanding of the cellular processes activated by the radiation.

The results obtained in the aforementioned research are listed in Table 1.

## 3. Radiation Effects on Cell Adhesion

Cell adhesion is a very convoluted process that requires several molecular procedures, such as alterations in the intercellular signalling pathways and the reorganization of the CSK. Cells adhere to the ECM through the formation of FAs, which requires actin polymerization. In particular, actomyosin contractions regulate both FAs structures and dynamics, therefore they can affect cell spreading, adhesion, and migration. Thus, FAs developed during cell spreading organise both the cytoskeletal architecture and the signals involved in the adhesion process [95,96]. Therefore, cell adhesion can be affected by a plethora of factors, including proteins and changes in the CSK organization.

### 3.1. Radiation-Induced Changes in the Cell Adhesiveness through the Activation of Proteins Pathways

Studies have shown that cells adhere to the different proteins forming ECM, such as fibronectin (FN), laminin (LN), and collagen (Col), through integrins receptors, which are primarily involved in the cell-ECM crosstalk [97]. In particular, FN can interact with some fibronectin-binding receptors, such as α5β1 integrin, implicated in the activation of a pathway responsible for the formation of FAs and, as a consequence, the cell adhesion process [98]. Lee and colleagues investigated the role of integrins in the regulation of radiation-altered adhesion between breast cancer cells, MDA-MB-231, and ECM proteins [99]. Ionizing radiation promotes their adhesion via the increase in the connections between malignant cells and FN. The reduction of the adhesion, stimulated by the radiations and the activation of α5β1 and α2β1 integrins, can be achieved through the use of the antibody against either of these receptors [99,100].

A crucial element for cell motility is the focal adhesion kinase (FAK), a protein implicated in the cell cycle, survival, and migration [101]. The formation of binding between cells and ECM activates the FAKs, which create a signalling complex with Src protein tyrosine kinase [102]. This complex, in turn, activates additional kinases, generates invadopodia and, therefore, leads to an increase in cell invasiveness [100,103,104,105,106]. In addition to this, overexpression of FAKs level results in some cancer types, such as glioblastoma or breast tumour [107]. It has been demonstrated that FAK-mediated formation of lamellipodia and invadopodia can increase cell motility through the stimulation of integrins, whose expression is increased by ionizing radiation. Some authors demonstrated that treatment of glioblastoma cells with the FAK inhibitor reduced adhesion by almost 20%, whereas in combination with a radiation dose of 4 Gy, the amount of attached tumour cells decreased by a further 5 to 10%. In the case of MDA-MB-231, the adhesion also decreased by about 50% after treatment with the inhibitor [108]. Nguemgo Kouam et al. showed that radiation treatment enhanced the activity of FAK and Src, which stimulated integrins and other proteins involved in the adhesion process, in both glioblastoma and breast cancer cell lines [109].

Another important role in the adhesive abilities of cells is played by the urokinase plasminogen activator surface receptor (uPAR), or CD87, which is involved in the interaction between integrins and the ECM, indirectly regulating cell adhesion [110]. Ionizing radiation increased the activation of the uPAR/integrin and β1/FAK pathway, enhancing the phosphorylation of cell adhesion-related molecules. Indeed, the uPA receptor (uPAR) binds uPA and vitronectin and is a co-activator of several integrins which leads to their interaction with ECM. So, indirectly, uPAR has an effect on cell-to-ECM adhesion and is implied in cell invasion. Nalla et al. demonstrated an increase in uPA and uPAR levels in two medulloblastoma irradiated cell lines, but also found their expression increased in non-irradiated invading cells [111]. Other researchers investigated the correlation with uPAR activation in an ex vivo study with the metastatic incidence of R-18 melanoma cell line engrafted in mice. Although the uPAR level increased after day 40, similar results were found for both control and irradiated tumour [112].

Another element plays an interesting role in the alteration of the cellular adhesion after the radiation treatment: the protein RhoA [113,114]. Ionizing radiation can affect cell adhesion to FN through the activation of RhoA/ROCK signalling pathways since they can control the FA assembly. This effect was proven in some studies, which have shown that RhoA is rapidly activated by a single high-dose of radiation, leading to RhoA/ROCK-dependent actin CSK remodelling [115] and that GTPase RhoA is involved in the molecular signalling involved of early endothelial responses to radiation as increased vascular permeability [68].

### 3.2. Alteration in Cells Adhesive Capabilities Stimulated by the Radiation-Induced Changes in the CSK

Several studies have investigated the changes in the CSK that affect cells’ ability to adhere to the ECM after the radiation treatment. For instance, the study performed on two different fibroblast cell lines, BALBc/3T3 and SVT2, showed that ionizing radiations altered the CSK structure, which, in turn, modified cells’ adhesive abilities. It was found that in control condition the cancerous cell line exhibited a disorganized actin CSK and a reduced adhesion in comparison with normal cells, after irradiation cell adhesiveness increased for both cell lines as evidenced by the enhancement of both spreading areas, FAs size, and cell stiffness [69,116,117,118]. Another study has investigated the alterations in the adhesion abilities of two mammary cell lines, MCF10A and the tumours counterpart MDA-MB-231, before and after the delivery of two doses of X-rays (2 Gy and 10 Gy) [119]. Cells were seeded on two polyacrylamide (PAAm) substrates with different stiffness, 1.3 kPa and 13 kPa, to mimic respectively the mechanical properties of healthy (soft) and cancerous (stiff) tissues. The study reported that the adhesiveness of the non-irradiated cancerous cell line on the softer substrate appeared higher than that of the healthy cells. This effect is due to the augmentation of the formation of FAs which is linked to the increase in contractility in KRAS-mutated cells [120]. After the delivery of both doses, MCF10A cells showed a decrease in their adhesive capabilities on both substrates 24 h after irradiation. In particular, this reduction resulted in inverse proportionality to the delivered dose for cells seeded on the stiffer substrate. The results reported this effect to be reversible for higher doses since cell adhesion values returned to the control condition 72 h after the radiation treatment. Conversely, MDA-MB-231 cells showed a decrease in their adhesiveness only when cultured on the softer substrate and irradiated with 2 Gy. On the other hand, the opposite phenomenon was observed for cells seeded on the stiffer substrate. These results proved that the healthy cell line had a higher ability to preserve its adhesive capabilities after the radiation treatment, probably due to a protection system put into effect by the mechanical microenvironment [119].

All the effects on cell adhesion described in the prior paragraph are listed in Table 2.

## 4. Radiation Effects on Cell Migration

Migration is a fundamental process for the preservation of the cellular organization [121]. Additionally, it is crucial for both physiological processes, including embryonic development, tissue remodelling and wound repair, and pathological phenomena, among which tissue fibrosis, tumour, and metastasis formation [122,123]. Migration involves the protrusion of the cell plasma membrane through the polymerization of the actin filaments, which are stabilized by FAs. In fact, cell motility is strongly regulated by processes of assembly and disassembly of FAs, which are in turn controlled by FAKs [124]. In particular, it was demonstrated that FAK signalling is associated with the disassembly of integrin-based adhesion sites and its expression level strongly increased in numerous human tumours [106]. The overexpression of FAK level in tumoral cells seems also to be responsible for the formation of invadopodia and podosomes, which lead to an invasive phenotype [108,125,126]. Several studies have investigated the outcomes of ionizing radiation on cell migration, mainly how radiation treatment can enhance cell motility via the rearrangement of the CSK or the expression level of adhesion proteins.

### 4.1. Ionizing Radiation Increase Cell Motility through the Alteration of the CSK

Changes in the CSK, induced by the radiation treatment, can affect several biological processes, among which cell migration. As previously stated, a plethora of work has demonstrated the existence of a strong correlation between the aggressive phenotype of cancerous cell lines and the changes in the cytoskeletal architecture [126,127]. Starting from this premise, some studies have been conducted to evaluate the ability of the radiation treatment to affect cell migration and to correlate these effects with changes induced on the CSK structure [128,129,130]. For instance, Panzetta et al. investigated the effects of different doses of radiation treatment on two fibroblast cell lines, BALBc/3T3 and SVT2. Their study showed that 24 h and 72 h after irradiation the healthy cell line, BALBc/3T3, reduced its speed and motility with both doses of 1 and 2 Gy [69]. This effect, more evident, was observed at higher doses too, after the delivery of 4 and 8 Gy [116]. Seventy-two hours after the radiation treatment, BALBc/3T3 cell motility returned to control levels, but only with a dose equal to 1 Gy. At higher doses, this cell line continued to show a decrease in its motility. The cancerous cell line, SVT2, showed similar behaviour, that is a reduction in its migratory abilities regardless of the doses. Considering that cell motility is a very sophisticated process based on a repeated cycle of membrane protrusion, attachment to the ECM, CSK contraction, and rear detachment from the ECM, tightly controlled by the FA life cycle (assembly–maturation–disassembly), the authors evaluated the impact of radiation on CSK and FAs’ components. In particular, they observed an increase in the organization and polymerization of actin filaments and to a consequent stiffening of the CSK, as supported by particle tracking microrheology (PTM) and atomic force microscopy (AFM) experiments [69,70,116,117]. As expected, the CSK stiffening was associated with the formation of longer FAs, which reduce cell migration speed because of their greater lifetime [131].

In another study, they investigated the radiation-induced alterations in the motility of two breast cell lines seeded on PAAm substrates [119]. The migration velocity of the non-irradiated healthy cell line, MCF10A, decreased with the increase in substrate stiffness. Indeed, it has been shown that cells seeded on stiff substrates can develop bigger stress fibres, which cause a more structured CSK, effectively slowing cell migration due to a decrease in the assembly/disassembly rate of FAs, as previously stated [131]. Regarding the cancerous cell line, MDA-MB-231, the increase in its motility was directly proportional to the increase in the stiffness of the substrate. This effect was observed not only in breast cells but also in other cancer cell lines, such as pancreatic and colorectal cells [132,133,134,135,136]. 24 h after the delivery of two doses of X-rays, equal to 2 and 10 Gy, MCF10A migration abilities increased on both substrates when irradiated with the lower dose, whereas the highest one did not seem to affect their motility. Conversely, 72 h after the radiation treatment the healthy cell line showed a decrease in its migratory abilities, especially on the softer substrate. Ionizing radiations affected the cancerous cell line by increasing their migration velocity on the softer substrate 24 h after irradiation, whereas the opposite phenomenon was observed 3 days after the treatment. On the other hand, the higher dose decreased cells’ motility on the soft substrate in a time-dependent manner. MDA-MB-231 cells seeded on the stiffer substrate showed a decrease in their motility only 72 h after irradiation, while 1 day after the treatment no change in their migration was observed [119]. The authors argued the possible existence of a radio-protective role of physiological ECM able to inhibit cell migration and invasion. After all, Cordes et al. had formerly reported a chemo- and radio-protective effect of some ECM molecules, observing an inhibited invasion of irradiated glioblastoma A-172 cells, probably due to an improved β1 and β2 integrin-mediated adhesion to FN and Matrigel [137]. However, further investigations are necessary to understand the molecular mechanisms governing cell responses to radiation.

Another study investigated the changes in the migratory and invasive capabilities of TSCC cells (Tca-8113) after the delivery of different doses of X-rays (0–4 Gy) [93]. Twenty-four hours after treatment, the wound-healing assay showed an increase in cell migration in a dose-dependent manner. Additionally, the Matrigel assay was used to study the invasive potential of this cell line. The results showed that the invasiveness of Tca-8113 cells increased together with the delivered dose. The augmentation in the migratory abilities of this cell line is motivated by the alteration in the cytoskeletal structure following irradiation since cells showed a depolymerization of the actin filaments, and, consequently, a decrease in their elastic modulus [93]. Jasińska-Konior et al. also observed this effect, as the disorganization of the actin CSK was involved in the alteration of the cell elasticity [66]. Other studies have investigated the correlation between cell elasticity and cell migration. It is known that a lower Young’s modulus can aid in cell invasion [138] since a softer cell can be easily deformed and is more likely to overcome tissue barriers [139,140].

### 4.2. Radiation-Induced Cell Migration through Protein Expression

The strong interaction existing between cell migration and the expression level of a large plethora of proteins has been elucidated. In particular, there are three different protein families that are analysed in the study of the radiation-induced effects on cell migration: integrins, small GTPases, and the LINC complex. Therefore, our discussion will focus on the effects induced by radiation on these three families.

Integrins are cell surface receptors that can identify ECM and cell-surface ligands [141]. These proteins regulate several biological functions, such as cell migration over ECM substrates, and the formation of adhesive junctions with the ECM [141,142,143]. Integrins are connected to the CSK through a multi-protein adhesion complex that links the ECM to the actin filaments. This connection grants the necessary forces to control shape change during cell migration [144].

It has been demonstrated that ionizing radiations can modulate the expression of integrins and, consequently, their migratory ability [137,145,146]. Nevertheless, it is important to highlight that the relationship between the expression level of integrins and cell migratory ability cannot be sharply defined. In fact, some studies indicate that the enhanced expression level of integrins (in particular α5β1) can act as a tumour suppressor by depleting cell migration and tumorigenicity [147] and, by contrast, some others report that integrin down-regulation enhances malignancy [148]. Consequently, the studies on the possible effects of radiation on the expression level of integrins and on the resulting tumour invasiveness, lead to very different conclusions. In particular, on one side, it has been reported that radiation induced the over-expression of β1 and β3 integrins in glioblastoma cells [137] and of β1, α2, α5, and α6 integrins in colon cancer cells [100]. The overexpression of integrin levels was associated in both cell lines with improved cell adhesion and in glioblastoma cells with an inhibited migration. On the other side, the enhanced expression level of β1 and β3 integrins has been reported to be involved in the activation of MMP-2 and MMP-9 enzymes that promote cell migration through the degradation of the ECM [137,145,149]. Another study reported the involvement of α3β1 integrin in the radiation-induced migration of meningioma cells. Specifically, several studies have demonstrated that α3β1 integrin is involved in the increase in cells’ invasive abilities [150,151]. The data showed that 24 h after treatment, the expression level of this integrin was enhanced and an increase in cells’ motility was observed [152]. In addition to this, another study proved that sublethal doses of ionizing radiations can increase glioma cells’ migratory abilities due to the expression of α5β3 integrin, but not α5β1 integrin, effectively implying that radiation-induced migration requires some specific integrins [153].

The small GTPases family is a protein family involved in almost all cellular processes [154]. One of the most important members of this family is the Rho GTPases family, which includes some fundamental proteins such as Rac1, RhoA and Cdc42. These proteins have the ability to control rearrangements of the cytoskeletal structure, cell cycle, and gene expression [155,156,157]. In particular, Rac1 is one of the most important regulators of the actin rearrangement and it controls cell cycle, adhesion and migration [158,159]; the protein RhoA is known to stimulate cell proliferation and invasion [160,161], and its overexpression is often found in cancerous cell lines [162,163]; lastly, the Cdc42 protein regulates cell proliferation, survival, and invasive capabilities [164,165,166].

In particular, while some studies have proven that RhoA GTPases can enhance cell motility, others have shown that high levels of this protein can reduce migration and stimulate adhesion [114,167]. Rousseau et al. observed the decrease in the migratory abilities of human microvascular endothelial cells (HMEC-1) after the delivery of 15 Gy, which led to a major increase in RhoA levels [115]. The inhibition of cell migration was observed in mast cells following LDIs. In particular, the decrease in cell motility was due to the rearrangement of F-actin, controlled by the P13K-Btk signalling pathway [168]. LDIs can induce a reduction in cell migration not only through the suppression of these proteins but also via the deactivation of the small GTPases Rac1/Cdc42. Additionally, the nuclear receptor Nr4a2, which is activated by cytokines and controls several functions such as proliferation and apoptosis [169], can increase cells’ migratory abilities through the triggering of the P13K-Btk signalling pathway [170,171,172,173]. Song et al. reported that LDIs reduce mast cell migration through the suppression of the monocyte chemoattractant protein-1 (MCP-1), which is a Nr4a2-regulated cytokine [174].

Finally, another important group of proteins that can influence cell migration is the LINC complex, which develops a direct connection between the CSK and the nuclear interior [175,176,177]. This complex is composed of the Sad1-UNC-84 (SUN) homology domain proteins interacting with KASH (Klarsichet/Anc1/Syne1 homology). Several studies have proven that the LINC complex can influence cell migration [178,179,180]. These proteins, in fact, together with the CSK control cell polarization and nucleus positioning in the cell rear define a leading-edge/centrosome/nucleus axis in the direction of migration.

Further, it has been demonstrated that the migratory abilities of several somatic mammalian cells are strongly regulated by the expression of SUN1 and SUN2 [181,182]. A study performed on the MDA-MB-231 cell line showed that, after the delivery of a sublethal dose of X-rays, both these proteins were necessary for the radiation-induced migration of cells. The results proved that ionizing radiation could trigger the alteration of the components of the LINC complex, effectively stimulating cell migration and invasion [183,184].

The effects of ionizing radiation on cell migration are listed in Table 3.

## 5. Conclusions and Future Perspectives

Changes in the CSK can lead to malignant transformation, during which a rearrangement of the actin CSK is observed [31,46]. In addition to this, alterations in the cytoskeletal architecture are connected to several modifications in cells’ properties, such as lower adhesive capabilities, associated with the structure of the CSK and the concentration of actin filaments and an increase in cells’ invasiveness [34,35]. Therefore, the study of the alteration of the CSK is fundamental for the comprehension of both tumorigenesis and invasive procedures.

**Figure 1:**
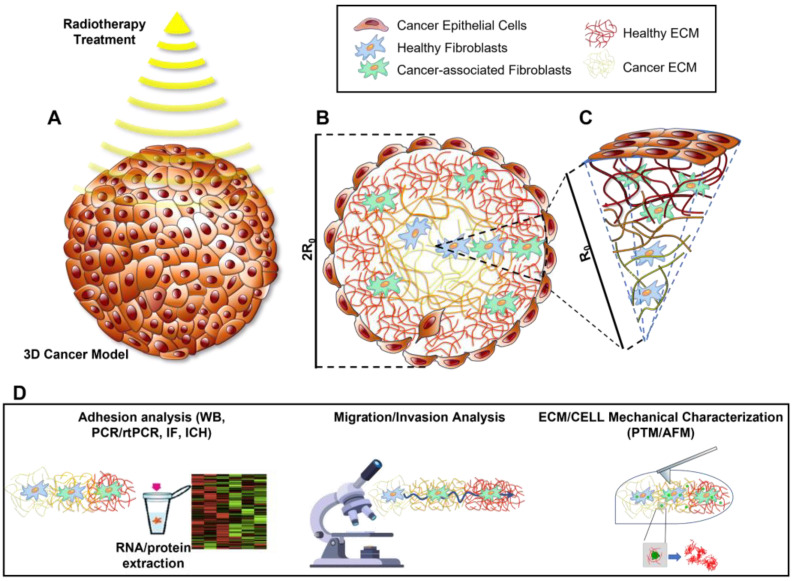
Schematic cartoon of 3D tissue-equivalents, able to resemble the major features of normal and tumour tissues at both cellular and extracellular level. 3D cancer model subjected to radiation therapy (**A**), cross-section (**B**) and spherical sector (**C**). Before and after irradiation, adhesion/migration/invasion analysis and ECM/cell mechanical characterization by PTM and AFM can be performed (**D**).

This brief review analyses different studies which have examined the radiation-induced alterations at the CSK scale. Although highly complex and scattered, the relationship between radiotherapy and CSK dynamics is clear. The collected results indicate, above all, a strong cell line dependence of radiation effects on the CSK and its functions, among which adhesion and migration. Furthermore, even though the cell specificity is the basis of cell biology, the great differences among these studies can be ascribed to many other reasons: (i) the very different time frames analysed, ranging from a few hours [61,71,124,125] to different days [48,77,82,83], can strongly affect cell responses; in particular, it has been reported that the response to radiations, in terms of adhesion and migration, can be completely reversed in a few days [109,113,124]; (ii) the single or collective cell model systems (wound-healing assay [87,93], Matrigel assay [59,87,93], single-cell migration [108,109,113], etc.); and (iii) the micro-environmental conditions (plastic Petri dish, protein-coated Petri dish, mimicking-tissue substrates). On this last matter, a large body of literature still discloses the central role of ECM in controlling cell behaviour [132,133] and tumour cell transformation [32,120], and recent works have revealed the importance of the microenvironment in mediating the cellular response to a physical insult such as photon radiation [52,53,54,119]. Considering all the above, it is becoming increasingly clear the importance to use 2D/3D biological tumour models that closely mimic the complexity and the heterogeneity of the native tumour microenvironment in terms of biomolecular composition, cellular population, tissue mechanics, and microarchitecture (Figure 1), [47,185]. At the same time, it will be also important to standardize models’ conditions during the experimental campaigns to guarantee a greater uniformity of the measurement methods. In particular, these sophisticated models will give the opportunity (i) to unveil more profoundly the intricate reciprocal relationship between tumour microenvironment and tumour cell status and stage and to study the effect of radiation on tumour tissues; (ii) to explore the effects of radiation on the architecture and mechanical properties of ECM, and (iii) correlate them to potential changes in cell metastatic and invasive grade. By pursuing this line, the understanding of the biophysical properties of the cell and ECM could lead not only to the innovation of the diagnostic tools but could also improve the existing cancer radiation treatments. Indeed, the importance of the selectivity/calibration of radiation therapy in producing distinct cellular responses in terms of total dose and treatment regime (accelerated fractionation, hyperfractionation, hypofractionation) could be investigated and this knowledge could be used in a preclinical/clinical context to provide an optimization protocol of their therapeutic outcome.

## Figures and Tables

**Table 1:** Effects of radiation on actin CSK.

Cell Line	Dose (Gy)	Time after Irradiation	Observed Effect on Actin CSK	Ref.
Mel270, BLM	1–3	40 days	Increase in marginal actin filaments and decrease in internal ones	[66]
BALBc/3T3 SVT2	1, 2	24 h	Actin polymerization, increase actin filaments	[69,70]
HUVEC	2–8	n.a.	Remodelling of the actin CSK	[72]
LN229 U87	2	20, 40 h	Activation of small GTPases Rac1K, increase in G-actin, decrease in F-actin	[77]
Cortical neurons	2, 4	24 h	Decomposition and rearrangement of the F-actin	[79]
Calu-3 16HBE14o-	2–10	4 h	Increase in F-actin depolymerization	[88]
MC3T3-E1	0.5, 5	5 days	Decrease in F-actin expression, expression of RhoA, ROCK1, and p-cofilin due to actin depolymerization	[89]
Murine exorbital lacrimal gland cells	0.036	4, 8 h	Actin depolymerization, increase in the cellular area (the outcomes were reversible after 24 h)	[92]
TSCC	0–4	24 h	Disorganization of the F-actin	[93,94]

**Table 2:** Effects of radiation on cell adhesion.

Cell Line	Dose (Gy)	Time after Irradiation	Observed Effect on Cell Adhesion	Ref.
MDA-MB-231	10	24 h	Increase in the connection between cells and FN	[99]
U-87 MG U-373 MG MDA-MB-231	0, 2, 4, 8	24, 48 and 72 h	Increased cell adhesion due to the activity of FAK and Src	[109]
HMEC-1	15	15 min	Increase cell adhesion due to FAs formation through the activation of RhoA/ROCK signalling pathways	[114,115]
BALBc/3T3 SVT2	1, 2, 4, 8	24, 72 h	Increased adhesion	[69,116,117,118]
MCF10A	2, 10	24 h	The decreased adhesion resulted in inverse proportionality with the delivered dose. (The effects were reversible after 72 h)	[119]
MDA-MB-231	2, 10	24, 72 h	Decrease adhesion with lower dose on the softer substrate, the opposite phenomenon was observed on the stiffer substrate	

**Table 3:** Effects of radiation on cell migration.

Cell Line	Dose (Gy)	Time after Irradiation	Observed Effect on Cell Migration	Ref.
BALBc/3T3, SVT2	4, 8	24, 72 h	Reduced speed and motility. (The effects were reversible after 72 h for BALBc/3T3)	[71]
BALBc/3T3, SVT2	1, 2	6, 24 h	Reduced speed and motility	[117]
MCF10A	2, 10	24, 72 h	After 24 h cells showed an increased motility with 2 Gy; 72 h after treatment cells showed a reduced motility	[119]
MDA-MB-231	2, 10	24, 72 h	After 24 h cells showed an increase in the migration velocity (this effect was reversible after 72 h)	
TSCC (Tca-8113)	0–4	24 h	Increase in cell migration in a dose-dependent manner	[93]
U251, U87	0–10	24 h	Increase in cell migration due to the expression of MMP-2 and MMP-9 enzymes	[137,149]
IOMM-Lee, CH-157-MN	7	24 h	Increase in cell motility due to the overexpression of α3β1 integrin	[152]
NIH-3T3	1–8	21 days	Increase in cell migration due to the expression of 𝛼5β3 integrin	[153]
HMEC-1	15	15 min	Decrease in cell motility	[115]
RBL-2H3	0.01, 0.05, 0.1, 0.5	N.A.	Decrease in cell migration through the suppression of the MCP-1	[174]
MDA-MB-231	0.5	24, 48 h	The expression of SUN1 and SUN2 proteins was necessary for the radiation-induced migration of cells	[184]
